# SIRT5 functions as a tumor suppressor in renal cell carcinoma by reversing the Warburg effect

**DOI:** 10.1186/s12967-021-03178-6

**Published:** 2021-12-20

**Authors:** Liu Yihan, Wang Xiaojing, Liu Ao, Zhang Chuanjie, Wang Haofei, Shen Yan, He Hongchao

**Affiliations:** 1 Department of Biostatistics, School of Public Health, Yale University, New Haven, CT 06520 USA; 2 Department of Urology, Shanghai Ruijin Hospital, Shanghai Jiaotong University School of Medicine, Shanghai, 200025 China; 3 Research Center for Experimental Medicine of Ruijin Hospital, Shanghai Jiaotong University School of Medicine, Shanghai, 200025 China

**Keywords:** SIRT5, Desuccinylation, PDHA1, Warburg effect, Renal cell carcinoma

## Abstract

**Background:**

The aim of this study was to investigate the biological functions and underlying mechanisms of SIRT5 in clear cell renal cell carcinoma (ccRCC).

**Methods:**

SIRT5 expression data in The Cancer Genome Atlas Kidney Clear Cell Carcinoma (TCGA-KIRC) were selected, and the correlations between SIRT5 expression and various clinicopathological parameters were analysed. SIRT5 expression in ccRCC tissues was examined using immunohistochemistry. Stable cell lines with SIRT5 knockdown were established. In vitro and in vivo experiments were conducted to investigate the functional roles of SIRT5 in the cellular biology of ccRCC, including cell viability assays, wound healing assays, soft agar colony formation assays, Transwell invasion assays, qRT–PCR, and Western blotting. In addition, microarrays, rescue experiments and Western blotting were used to investigate the molecular mechanisms underlying SIRT5 functions.

**Results:**

SIRT5 expression was downregulated in ccRCC compared with normal tissues, which correlated with a poor prognosis of ccRCC. SIRT5 knockdown significantly increased cell proliferation, migration and invasion in vitro. In vivo experiments revealed that SIRT5 knockdown promoted ccRCC tumorigenesis and metastasis. Mechanistically, SIRT5 deglycosylated PDHA1 at K351 and increased PDC activity, thereby altering the metabolic crosstalk with the TCA cycle and inhibiting the Warburg effect. SIRT5 overexpression was related to low succinylation of PDHA1.

**Conclusions:**

Downregulated SIRT5 expression in ccRCC accelerated the Warburg effect through PDHA1 hypersuccinylation and induced tumorigenesis and progression, indicating that SIRT5 may become a potential target for ccRCC therapy.

## Background

Renal cell carcinoma (RCC) is one of the most aggressive malignancies of the urinary system, and its morbidity has increased gradually in the past few years. According to US cancer statistical data, 65,000 newly diagnosed cases and approximately 15,000 deaths are reported annually [1]. Clear cell renal cell carcinoma (ccRCC), accounting for > 80% of the histopathological types of sporadic RCC, is associated with worse survival outcomes than other subtypes [2]. Metastatic ccRCC has a poor prognosis, and the 5-year overall survival (OS) rate is approximately 10%, while the 5-year OS rate of surgically treated stage I ccRCC is > 90% [3]. Although combination strategies, including surgery, targeted therapy, and immunotherapy, have optimized the overall efficacy of ccRCC treatment, the clinical efficiency is still limited, especially in metastatic ccRCC [4]. Therefore, investigations of the detailed molecular mechanisms underlying the tumorigenesis of ccRCC and novel therapeutic strategies are urgently needed.

Tumorigenesis depends on the reprogramming of cellular metabolism, which permits sustained biomass accumulation and redox homeostasis from a frequently nutrient-poor environment [5]. Cancer cells primarily utilize glycolysis for energy generation, even in oxygen-rich conditions, and this phenomenon is known as the Warburg effect, a metabolic hallmark of cancer [6]. Pyruvate, the end product of glycolysis, is transformed into lactate in the cytoplasm or transported into mitochondria and catalysed by the pyruvate dehydrogenase complex (PDC), providing acetyl-CoA and NADH for the TCA cycle and biosynthetic processes [7–13]. PDC was also reported to translocate into the nucleus and generate acetyl-CoA for histone acetylation [14]. Eukaryotic PDC is composed of pyruvate dehydrogenase (E1), dihydrothioamide acetyltransferase (E2) and dihydrothioamide dehydrogenase (E3) [7]. Eukaryotic cells have evolved strategies to regulate protein activity through posttranslational modifications, such as phosphorylation, acetylation, and succinylation [15–20]. PDC activity depends on the typical phosphorylation state of PDHA1, which is phosphorylated by pyruvate dehydrogenase kinase (PDK) 1–4 and dephosphorylated by pyruvate dehydrogenase phosphatase (PDP) 1–2 [10–12].

SIRT3 deacetylates PDHA1 at K321, recruits PDP1 and finally increases PDC activity [21, 22]. Another mitochondrial SIRT4 hydrolyses the lipoamide cofactor from the E2 component dihydrolipoyllysine acetyltransferase (DLAT) and reduces PDH activity [23]. Recently, SIRT5, one of the three mitochondrial sirtuins, was reported to desuccinylate various proteins, such as PDHA1 and SHDB, and regulate the activities of these proteins [20, 24]. However, the relationship between SIRT5 and cancer metabolism in ccRCC remains elusive. In this study, we aimed to investigate the expression and potential role of SIRT5 in ccRCC.

## Methods

### Sample collection from patients with ccRCC

This study was approved by the Ethics Committee of Shanghai Ruijin Hospital, and all patients provided written informed consent. ccRCC specimens (n = 280) that were histopathologically confirmed by three independent pathologists were prepared as a tissue microarray with matched normal sections. Six paired tumor and adjacent normal tissues were also obtained from patients with ccRCC who underwent radical nephrectomy in our institution. Clinical information was obtained from the medical records, including age, sex, tumor size, grade, and stage. Tumor samples and adjacent normal tissues were placed in liquid nitrogen followed by storage at −80 °C. In addition, computed tomography (CT)-guided biopsies were collected from two patients with ccRCC, and patient-derived organoids (PDOs) were established and cultured.

### Cell culture, materials and antibodies

HEK293T cells (ATCC CRL-11268) were cultured in DMEM (Gibco 12430-054) containing 10% FBS (Gibco 10099141), HeLa cells (ATCC CCL-2) and KMRC-20 cells (JCRB JCRB1071) were cultured in MEM (Gibco 11095-080) containing 1% NEAA (Gibco 11140-050) and 10% FBS, and Caki-1 cells (ATCC HTB-46) were cultured in McCoy’s 5A medium (Gibco 11095-098) containing 10% FBS. 786 O cells (ATCC CRL-1932) and RENCA cells (ATCC, CRL2947) were cultured in DMEM supplemented with 10% FCS (Gibco 10500-064). Cells were transfected with plasmids using Lipofectamine® 2000 Transfection Reagent (Thermo Fisher Scientific Cat. No: 12566014). Wild-type SIRT5, SIRT5^Δ50^ (lacking the mitochondrial signal peptide, 1–36 AAs), wild-type PDHA1, and PDHA1 mutants were cloned into the pcDNA3.1-Flag, pcDNA3.1-Myc and pcDNA3.1-HA vectors, respectively. The SIRT5-specific shRNA (5’-GCTGGAGGTTATTGGAGAA-3’, 5’-CAGCATCCCAGTTGAGAAA-3’) was used to knock down SIRT5 expression, and the nonsilencing shRNA oligonucleotide was used as a negative control. The anti-pan-succinyl lysine antibody and anti-succ-K351 of PDHA1 antibody were generated in our lab. Synthetic PDHA1 peptides containing succinylated K351 were used as antigens to immunize rabbits, and purified serum proteins were measured. Antibodies specific to pyruvate dehydrogenase E1-alpha subunit antibody (Abcam), SIRT5 (Sigma), β-actin (Genscript), Flag-Tag (Abmart), Myc-Tag (Abmart), HA-Tag (Cell Signaling Technology), and mitochondria (Abcam) were purchased. PDH activity was determined using a PDH Enzyme Microplate Assay Kit (Abcam ab109902).

### In vitro succinylation and desuccinylation

We succinylated the lysine-containing peptides (GL Biochem) by mixing 0.5 mM succinyl-CoA (Sigma) and 50 ng/μl peptides in 30 mM HEPES (pH 7.4) in a 15 μl system and incubating them at 37 ℃ for 3 h. The peptides were desalted using C18 ZipTips (Millipore) prior to the MALDI-TOF MS analysis. Desuccinylation was carried out by incubating enzymes with SIRT5 (Sigma) at 37 °C for 3 h in 30 mM HEPES buffer (6 mM MgCl_2_, 1 mM DTT, and 1 mM NAD^+^, pH 7.4).

### Animal studies

Four-week-old male nude BALB/c mice obtained from Shanghai Lingchang Biotechnology Co., Ltd. were used to establish the orthotopic tumor model. Mice had ad libitum access to a standard diet and water. Cages were maintained in well-ventilated racks in a temperature- and humidity-controlled environment with a 12 h light/dark cycle. All animal experiments were approved by our institutional Animal Research Ethics Committee. Luciferase-expressing Renca cells (1 × 10^5^) stably transfected with shCtrl or shSIRT5 in 25 µL of 2:1 (v/v) PBS:Matrigel were injected into the subrenal capsule of the right kidney of BALB/c mice (5 mice/group). In vivo bioluminescence imaging (BLI) was performed to record the growth of tumors every 5 days, and Living Image® software was used to quantify the BLI signal. Mice were euthanized under anaesthesia 28 days after implantation, and tumor samples were fixed with 4% paraformaldehyde overnight, embedded in paraffin, and cut into 4 μm paraffin sections for subsequent experiments.

An in vivo lung metastasis model was generated using male nude BALB/c mice aged 4 weeks by a tail vein injection of Caki-1 cells stably expressing luciferase. Lung metastatic progression was monitored using the IVIS-100 system (Caliper Life Sciences). In vivo BLI was performed every 7 days, and Living Image® software was used to analyse the data. Mice were euthanized 28 days after implantation, and lung metastatic lesions were analysed using the BLI system.

### Western blot analysis

Western blot analyses were performed using standard procedures. After cells were harvested, the protein concentration was determined using Quantity One software (Bio-Rad, Hercules, Calif., USA). Cell lysates were separated on 10% SDS–PAGEPAGE gels, transferred to PVDF membranes (Millipore, Bedford, MA, USA) and blocked with 5% w/v skim milk for 2 h at room temperature. Anti-PDHA1, anti-Succ-K351 PDHA1, anti-SIRT5, anti-GAPDH, and anti-actin antibodies were incubated with the membrane overnight at 4 °C. Immune complexes were detected with a horseradish peroxidase-conjugated secondary antibody (1:3000, Southern Biotech) for 2 h at RT. Finally, bands were visualized using an enhanced chemiluminescence system (ECL; Pierce Company Woburn, MA, USA). Western blot signals were obtained by detecting chemiluminescence with a Typhoon fla9500 system (GE Healthcare). ImageJ software (NIH) was used to analyse the densities of the bands. Each blot shown in the figures is representative of at least three experiments.

### Immunofluorescence analysis

Standard procedures were used for the immunofluorescence analysis. Cells were seeded in 24-well plates, fixed with 4% paraformaldehyde, and then permeabilized with 1% Triton. Cells were incubated overnight at 4 °C with anti-PDHA1 and SIRT5 antibodies and detected the next day with an Alexa Fluor 555-conjugated goat anti-mouse IgG antibody. The nuclei were stained with DAPI (Sigma). Immunofluorescence images were observed with a fluorescence microscope (Leica, DMI4000B).

### Measurement of the activity of PDHA1 complex

PDHA1 activity was measured in a reaction buffer containing 50 mM KH_2_PO_4_ (pH 7.0), 1 mM MgCl_2_, 2 mM sodium pyruvate, 0.2 mM thiamine diphosphate and 0.1 mM 2,6-dichlorophenolindophenol (2,6-DCPIP). The purified PDHA1/PDHB complex was added to start the reaction. The reaction was maintained at 30 °C. The course of the reaction was monitored by measuring the reduction of 2,6-DCPIP at 600 nm using a Roche spectrophotometer.

### Oxygen consumption rate (OCR)

KMRC-20 cells or Caki-1 cells were seeded on XFe24 cell culture microplates (Seahorse Biosciences) at densities of 40,000 or 15,000 cells/well, respectively. The analysis was performed using the XF cell Mito stress test kit (Seahorse Bioscience) according to the manufacturer's protocol. The culture medium was replaced with assay medium (XF Base Medium containing 5.5 mM glucose, 2 mM glutamine, 1% FBS, 1 nM insulin, and 100 nM dexamethasone, pH 7.4) (Seahorse Bioscience) 1 h before the analysis. Oligomycin, FCCP, rotenone and antimycin A used in assays were at final concentrations of 2 μM, 1 μM, 1 μM and 1 μM, respectively. The results were normalized to the cell numbers.

### Measurement of the growth curves

PDHA1 knockout cells were transfected with SIRT5, PDHA1, PDHA1 + SIRT5, PDHA1^K351Q^ or PDHA1^K351Q^ + SIRT5 and seeded in 96-well plates. Cell morphology was observed under an inverted microscope, the plate and its contents were allowed to equilibrate at room temperature for approximately 30 min, the transparent bottom was affixed with a white back cover, and the luminescence was recorded with EnSpire.

### Soft agar colony formation assay

For soft agar colony formation assays, 2 ml of medium containing 10% FBS and 0.7% agar were used. Cells were seeded in 2 ml of medium containing 10% FBS with 0.35% agar at a density of 1 × 10^5^ cells per well and incubated for 21 days at 37 °C. Then, the number of colonies that formed in soft agar was counted using ImageJ software, and images were captured using an Olympus IX5 microscope.

### Wound-healing assay

Cell migration was assessed by performing a wound healing assay. Briefly, Caki-1 cells were transfected with PDHA1, PDHA1 + SIRT5, PDHA1^K351Q^ or PDHA1^K351Q^ + SIRT5. Approximately 5 × 10^6^ cells were seeded into 24-well plates and cultured for 24 h. Then, a yellow plastic pipette tip was used to create a wound by scraping the cells. Cell migration was monitored under a Nicon Eclipse microscope and photographed at 100× .

### Transwell invasion assay

Cell invasion experiments were carried out using 24-well Transwell plates with 8 μm pore size polycarbonate Matrigel-coated membrane inserts according to the manufacturer’s instructions. After 20 h of incubation, noninvading cells in the upper insert were removed, and cells that had invaded into the lower Matrigel surface were fixed with 4% paraformaldehyde and stained with 0.5% crystal violet. Ten random fields of view were quantified.

### Immunohistochemistry

Formalin-fixed and paraffin-embedded specimens were prepared for histological sectioning. Antigen recovery was performed on renal carcinoma specimens incubated with Tris–EDTA buffer (pH 8.4) at 99 °C for 60 min. Endogenous peroxidase activity was inactivated by incubating sections with a methanol and 3% H_2_O_2_ solution. Sections were incubated with the primary antibody for 60 min, secondary antibody for 8 min, and DAB developer for 8 min. All procedures were performed using the Ventana BenchMark XT automated stainer, and the sections were scanned using a Ventana iScanCoreo scanner. IHC results were quantified by experienced pathologists. The intensity was calculated according to the positive area and the degree of positive staining. The sections were stained with SIRT5 (1:100), PDHA1 (1:100), Succ-K351-PDHA1 (1:100) and Ki67 (1:100) antibodies using an ultraView Detection Kit.

### Statistical analysis

Data are presented as the means ± standard deviations (SD), and bars in the graphs represent standard deviations. Statistical analyses were conducted using SPSS 22.0 software (IBM, Corp., Armonk, NY, USA). Significant differences between groups were determined using Student’s t test. The significance level for statistical testing was set to *P* < 0.05.

## Results

### SIRT5 is downregulated and associated with a poor prognosis for patients with ccRCC

**Fig. 1:**
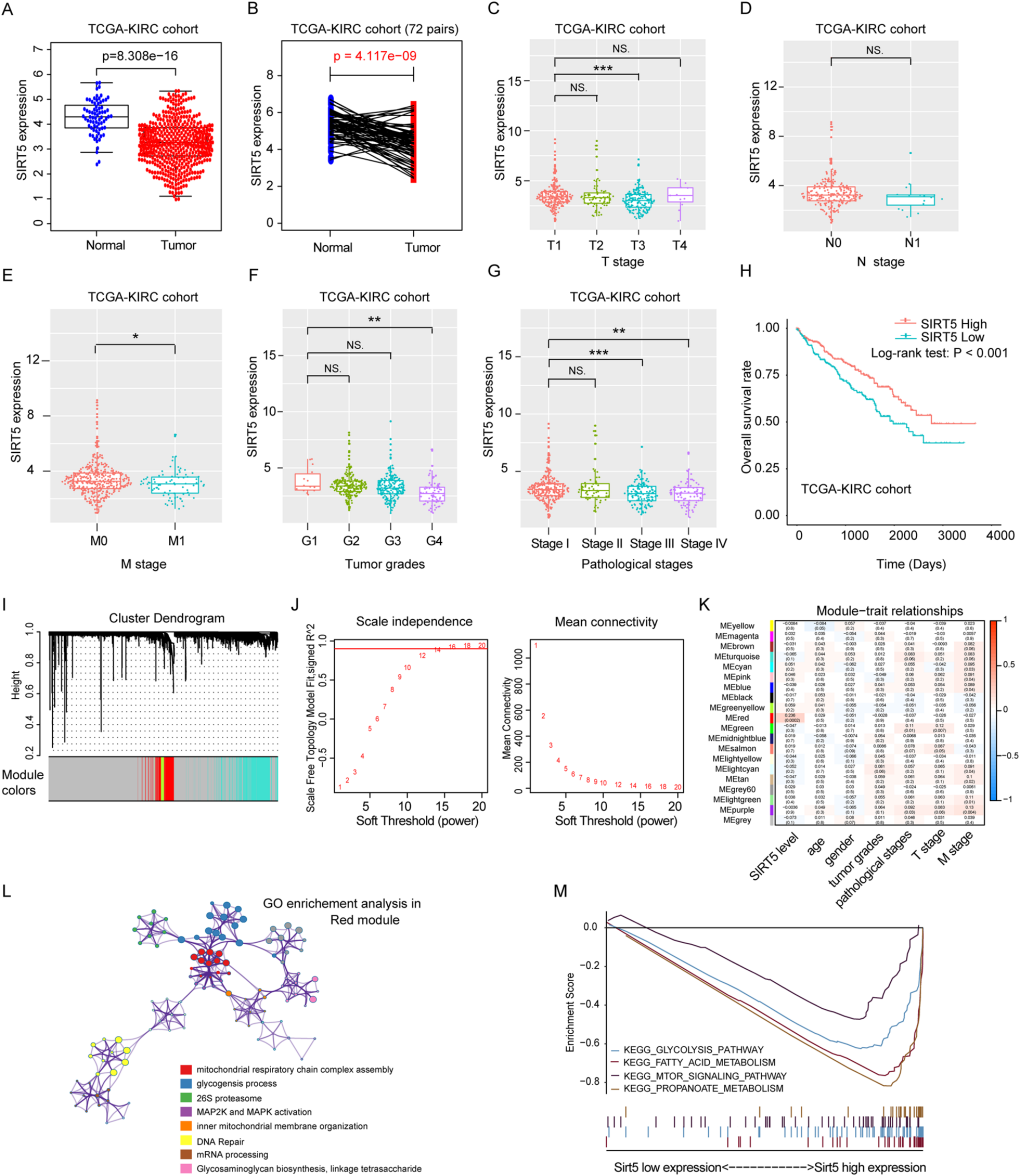
SIRT5 is downregulated and associated with the prognosis of patients with ccRCC. **A** SIRT5 expression in tumor and normal tissues in TCGA-KIRC dataset. **B** SIRT5 expression in 72 paired ccRCC tissues from TCGA-KIRC cohort. SIRT5 expression levels were compared in groups stratified by different clinicopathological parameters: TNM stage (**C**–**E**), tumor grade (**F**) and pathological stage (**G**). **H** Kaplan–Meier analysis of OS of patients stratified by SIRT5 expression in TCGA-KIRC dataset. Cluster dendrograms (**I**), soft thresholds (**J**) and module-trait relationships (**K**) were analysed using WGCNA. **L** GO functional enrichment analysis. **M** KEGG pathway analysis

The RNA-seq datasets and clinical information of patients with ccRCC were downloaded from The Cancer Genome Atlas (TCGA; https://portal.gdc.cancer.gov/). We performed a comprehensive bioinformatics analysis and found significantly lower SIRT5 expression in ccRCC tissues than in normal tissues (*P* = 8.308e^−16^) (Fig. 1A). We further detected the expression of SIRT5 in 72 paired ccRCC tumor samples obtained from Ruijin Hospital and found that SIRT5 expression was significantly decreased in the tumor samples compared with paracancerous normal tissues (*P* = 4.117e−09) (Fig. 1B). In addition, patients with ccRCC in TCGA-KIRC cohort with lower SIRT5 expression levels had a higher clinicopathological TNM stage and grade (Fig. 1C–G). The Kaplan–Meier analysis verified that patients with lower SIRT5 levels had a worse overall survival prognosis (Fig. 1H). We further performed a weighted gene coexpression network analysis (WGCNA) based on TGCA-KIRC samples and found that the correlation coefficient between SIRT5 levels and identified MEred modules was 0.236 (*P* = 0.0002) with a threshold of 12 (Fig. 1I–K). The GO functional enrichment analysis indicated that SIRT5 was primarily related to mitochondrial respiratory chain complex assembly and MAP2K/MAPK activation (Fig. 1L). The KEGG pathway analysis also indicated that SIRT5 participated in the fatty acid metabolism pathway and propionic acid metabolism pathway (Fig. 1M). Based on these data, we concluded that SIRT5 might be a tumor suppressor that is involved in ccRCC tumorigenesis by regulating cancer metabolism.

### SIRT5 desuccinylates PDHA1

**Fig. 2:**
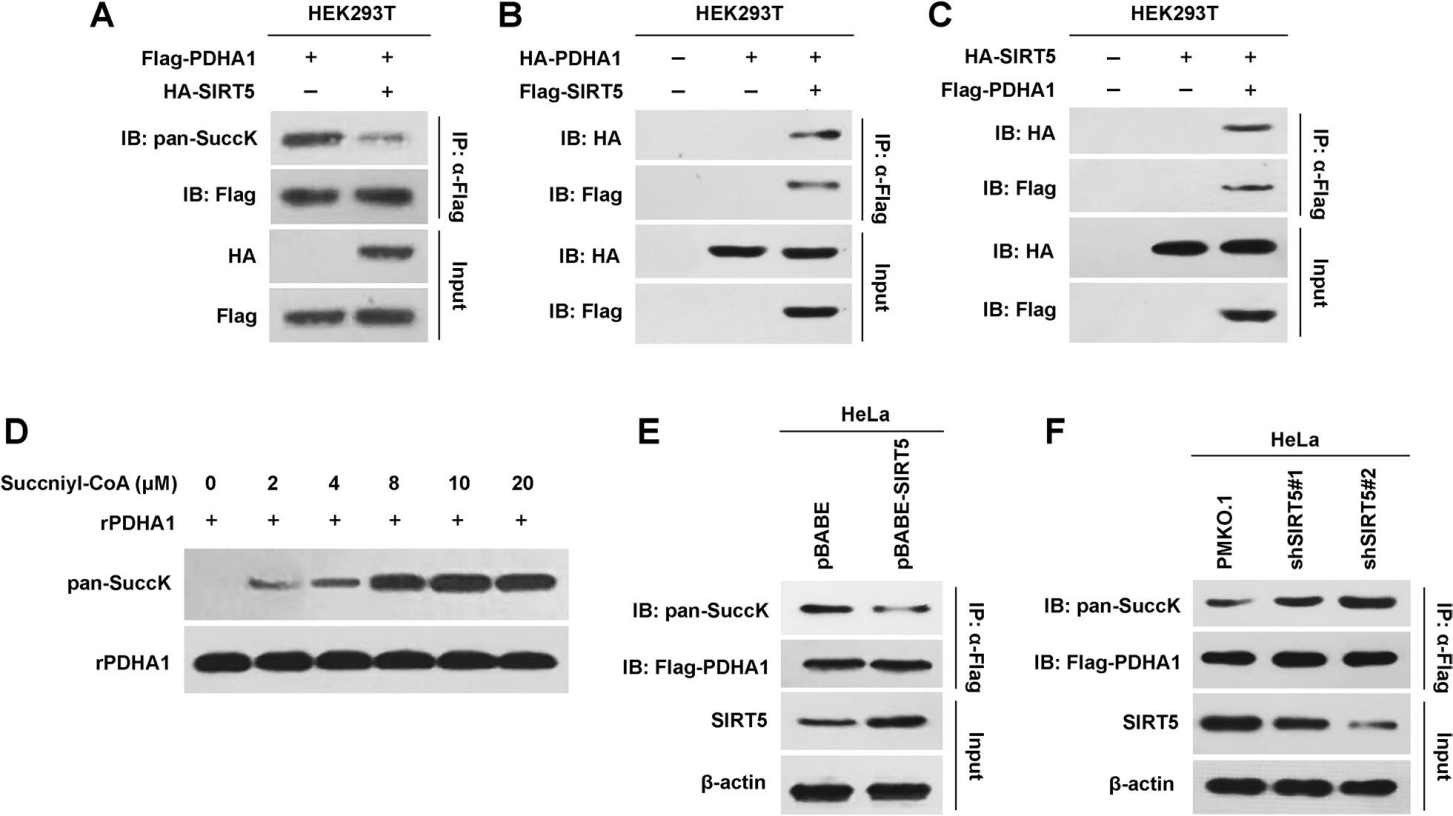
SIRT5 desuccinylates PDHA1. **A** Flag-PDHA1 and HA-SIRT5 were transfected into HEK293T cells, and the pansuccinylation level of PDHA1 was determined. **B** HA-tagged PDHA1 and Flag-tagged SIRT5 were coexpressed in HEK293T cells. PDHA1 copurified with SIRT5 was detected using an HA antibody. **C** HA-tagged SIRT5 and Flag-tagged PDHA1 were coexpressed in HEK293T cells. SIRT5 copurified with PDHA1 was detected using an HA antibody. **D** Diluted succinyl-CoA was added to the purified PDHA1 system, and the pansuccinylation level of recombinant PDHA1 (rPDHA1) was determined. **E** The pBABE-SIRT5 and control vectors were introduced into HeLa cells, and pansuccinylation of PDHA1 was determined. **F** SIRT5 was knocked down in HeLa cells, and pansuccinylation of PDHA1 was determined

Wild-type SIRT5 decreased the pansuccinylation of PDHA1 (Fig. 2A), suggesting that PDC activity might be regulated by SIRT5. We tested this hypothesis by first analysing the interaction between SIRT5 and PDHA1. SIRT5 was copurified with PDHA1, and PDHA1 was copurified with SIRT5 when they were coexpressed in HEK293T cells (Fig. 2B and C), suggesting that SIRT5 and PDHA1 interacted with each other. Incubation of recombinant PDHA1 (rPDHA1) with succinyl-CoA resulted in a gradual increase in the levels of succinylated PDHA1 in a succinyl-CoA-dependent manner (Fig. 2D), confirming that PDHA1 was succinylated directly. Moreover, SIRT5 overexpression removed the succinylation of PDHA1 in the cell line stably expressing SIRT5 (Fig. 2E), whereas SIRT5 knockdown increased PDHA1 succinylation (Fig. 2F). Thus, SIRT5 contributed to the desuccinylation of PDHA1.

### SIRT5 desuccinylates K351 of PDHA1

**Fig. 3:**
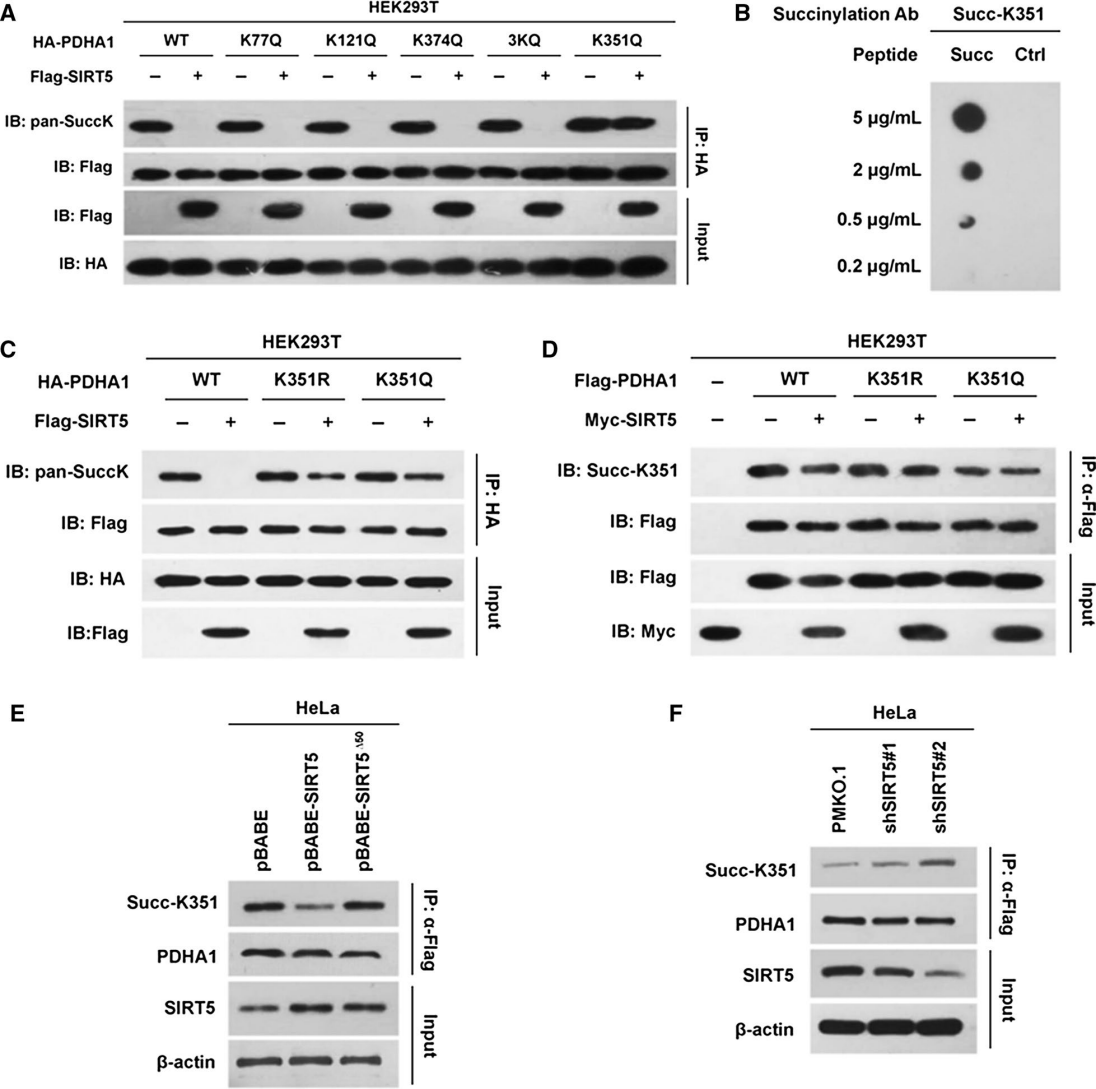
SIRT5 desuccinylates PDHA1 at K351. (A) Lysine 77 (K77), K121, K374, K351 and 3 K (K77, K121 and K374) of PDHA1 were mutated to glutamine and introduced into HEK293T cells. Then, the pansuccinylation level of PDHA1 was determined in the presence or absence of SIRT5. **B** Validation of the specificity of the Succ-K351 antibody. Succ-K351 represented the peptide containing succinylated K351 and the flanking sequence. **C** Lysine 351 (K351) of PDHA1 was mutated to arginine (R) or glutamine (Q) and then transfected into HEK293T cells with or without SIRT5, and the pansuccinylation level of PDHA1 was determined. **D** K351R and K351Q PDHA1 mutants were transfected into HEK293T cells with or without SIRT5, and the succinylation level of PDHA1 was determined. **E** SIRT5^∆50^ was transfected into HeLa cells, and the succinylation level of endogenous PDHA1 was determined. **F** The succinylation levels of endogenous PDHA1 in HeLa cells before and after SIRT5 knockdown by independent shRNAs were compared

We determined which lysine sites of PDHA1 were desuccinylated by SIRT5 by mutating each candidate lysine site of PDHA1 according to previously published data [15, 18]. SIRT5 had no effect on the succinylation of K77, K121 and K374 in PDHA1 but desuccinylated K351 in PDHA1 (Fig. 3A), suggesting that K351 in PDHA1 might be the substrate of SIRT5. Interestingly, SIRT5 only decreased the succinylation of wild-type PDHA1 but not its desuccinylation mimetic K351R or succinylation mimetic K351Q mutants, indicating that K351 might be the substrate of SIRT5 (Fig. 3C). We verified that K351 in PDHA1 is the substrate of SIRT5 by generating site-specific antibodies against Succ-K351 of PDHA1 using synthetic succinylated PDHA1 peptides as antigens (Fig. 3B). The levels of Succ-K351 in endogenous PDHA1 were measured following SIRT5 overexpression and knockdown to test whether SIRT5 desuccinylated endogenous PDHA1. PDHA1 was knocked down and then reintroduced into the HEK293 cell line to exclude a potential effect of endogenous PDHA1. SIRT5 decreased the K351 succinylation of wild-type PDHA1 but had no effect on the K351 mutants (Fig. 3D). Overexpression of wild-type SIRT5 in HeLa cells decreased the levels of endogenous Succ-K351 PDHA1; however, overexpression of the mitochondrial localization defective but functional mutant (SIRT5^∆50^) did not (Fig. 3E). Conversely, SIRT5 knockdown increased the level of Succ-K351 in endogenous PDHA1 (Fig. 3F). These results collectively supported the hypothesis that K351 in PDHA1 might be the site desuccinylated by SIRT5.

### SIRT5 desuccinylates PDHA1 and regulates metabolic pathways

**Fig. 4:**
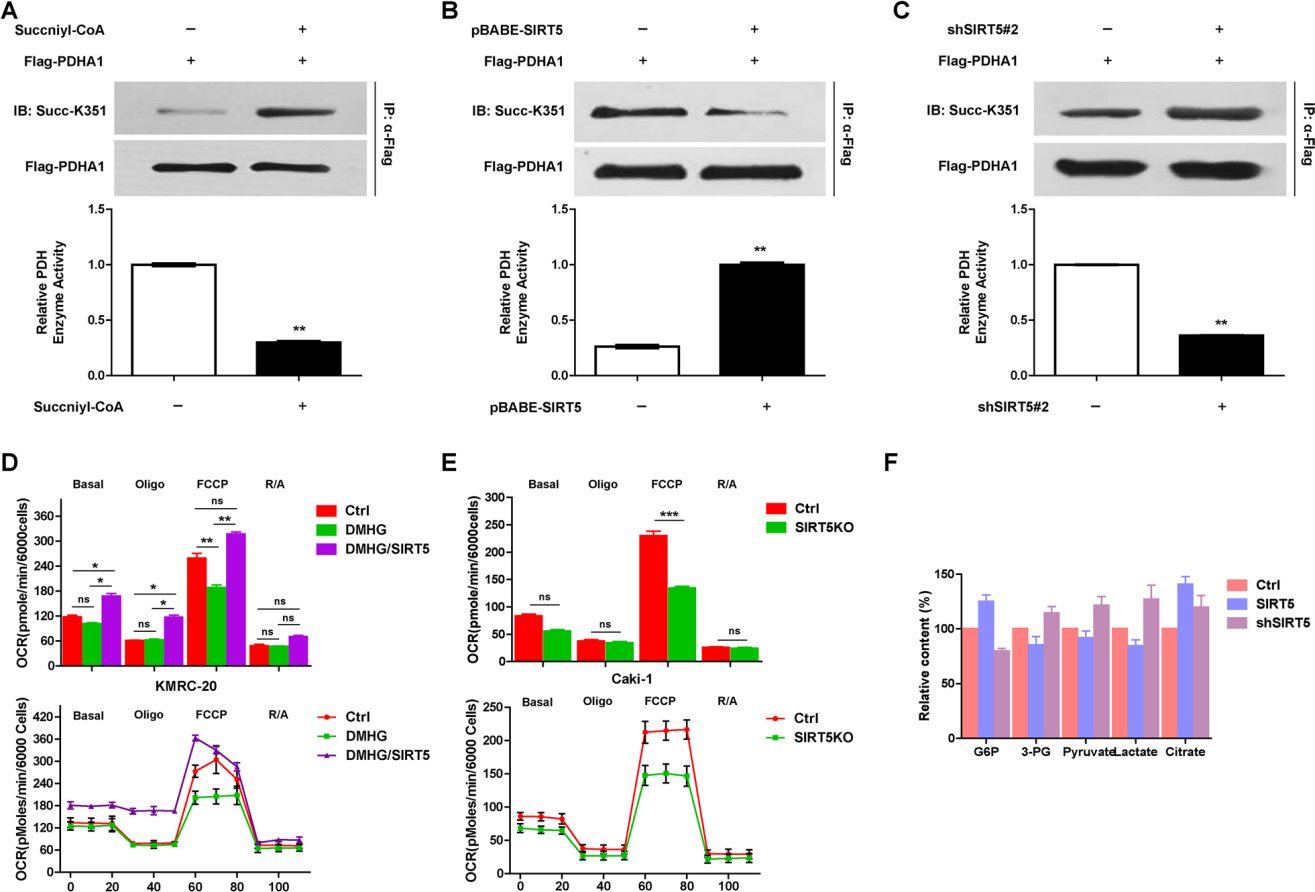
SIRT5 regulates PDHA1 activity and remodels metabolic pathways. **A** Succinyl-CoA was added to the PDHA1 system, and the levels of succinylated PDHA1 and PDHA1 enzyme activity were determined. The levels of succinylated PDHA1 and PDHA1 enzyme activity were determined following the overexpression (**B**) or knock down of PDHA1 (**C**). **D** OCRs were detected for untransfected and SIRT5-transfected KMRC-20 cells that were treated with DMHG. OCRs were recorded from cells treated with oligomycin, carbonyl cyanide-m-chlorophenylhydrazone (FCCP), and antimycin A/rotenone at the indicated time points. **E** SIRT5 was knocked down in Caki-1 cells, and the OCR was detected and quantified. **F** The levels of key metabolites produced by glycolysis and the TCA cycle were determined following SIRT5 overexpression or knockdown

We confirmed that SIRT5 desuccinylates PDHA1 and reroutes the metabolic pathways by first testing the effect of succinyl-CoA on the succinylation and activity of PDHA1. Succinyl-CoA increased the succinylation of PDHA1 but decreased its activity (Fig. 4A). Then, the succinylation and activity of PHDA1 were evaluated under conditions of SIRT5 overexpression or knockdown were evaluated. Overexpression of SIRT5 decreased PDHA1 succinylation but increased its activity (Fig. 4B). Conversely, SIRT5 knockdown increased PDHA1 succinylation and decreased its activity (Fig. 4C).

We further evaluated the OCR and metabolites of glycolysis and the TCA cycle to confirm that metabolic pathways were reprogrammed by SIRT5. The OCR of KMRC-20 cells was decreased upon treatment with 5 mM DMHG, but SIRT5 overexpression rendered the OCR of KMRC-20 cells resistant to DMHG (Fig. 4D), suggesting that SIRT5 regulated the OCR of KMRC-20 cells. Additionally, SIRT5 knockdown decreased the OCR of Caki-1 cells (Fig. 4E). Moreover, we analysed the metabolites of glycolysis and the TCA cycle and found that SIRT5 decreased the concentrations of glucose-6-phosphate (G6P), 3-phosphoglyceric acid (3-PG), pyruvate and lactate and increased citrate levels (Fig. 4F), indicating that SIRT5 decreased glycolysis and increased the TCA cycle.

**Fig. 5:**
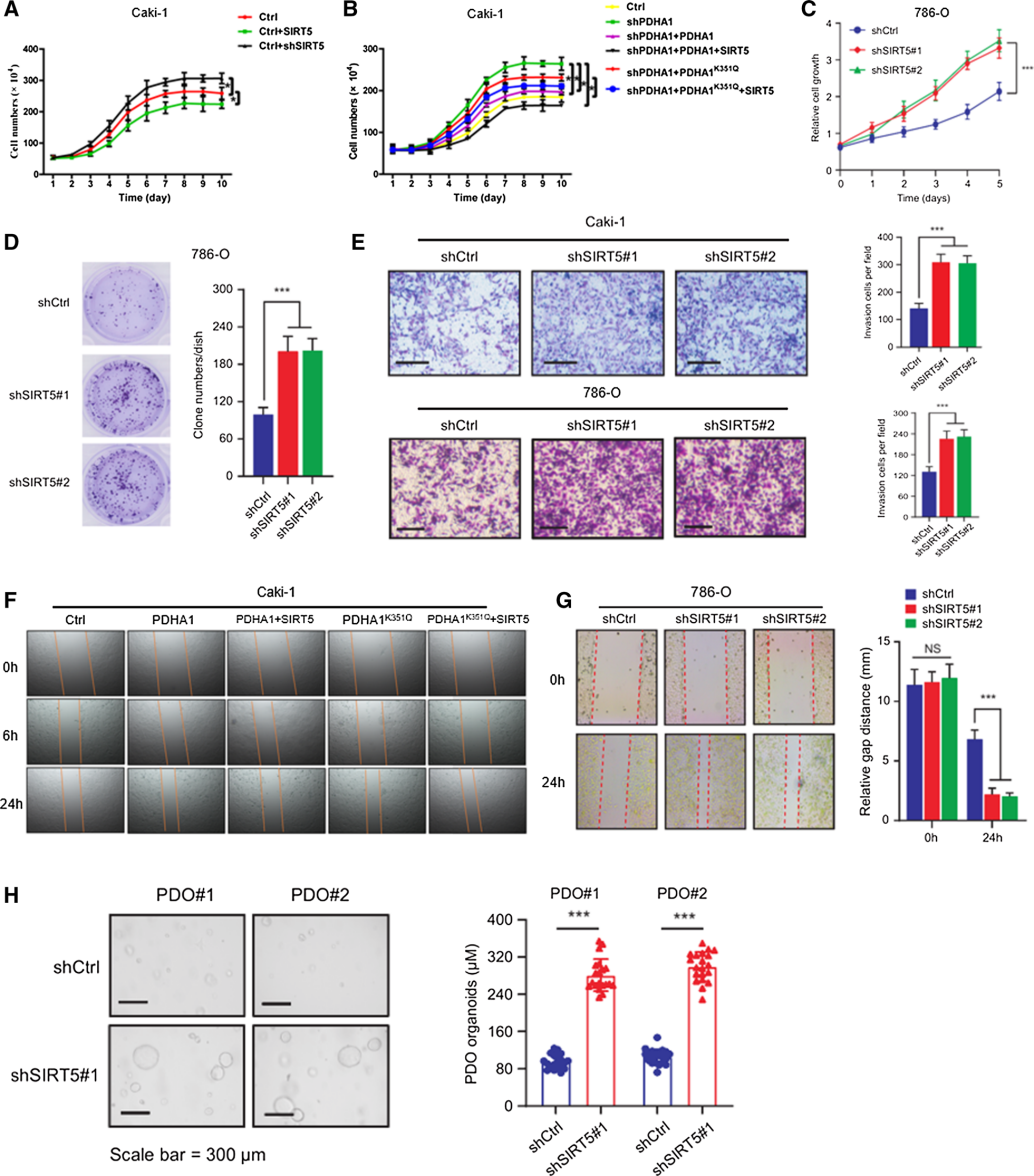
SIRT5 decelerates cell proliferation and inhibits cell migration through PDHA1. **A** Growth curves of Caki-1 cells overexpressing or silenced for SIRT5 were determined. **B** PDHA1 was knocked down in Caki-1 cells, and then the wild-type and K351Q mutant (PDHA1^K351Q^) PDHA1 plasmids were reintroduced into the cells. The growth curves of two modified Caki-1 cell clones with or without SIRT5 expression were compared. Growth curves (**C**) and (**D**) clone formation assays 786-O cells with SIRT5 knockdown were determined. **E** Transwell invasion assay using wild-type and shSIRT5-expressing Caki-1 and 786-O cells. **F** Wild-type and mutant PDHA1^K351Q^ PDHA1 were introduced into Caki-1 cells, and photos of cells with or without SIRT5 expression were captured at 0, 6, and 24 h. **G** Wound healing assay of SIRT5 knockdown in 786-O cells. **H** Representative images of two different ccRCC organoids transfected with the shCtrl or shSIRT5 lentivirus and quantification of organoid diameters

Last, SIRT5 overexpression dramatically decelerated Caki-1 proliferation, whereas SIRT5 knockdown significantly promoted Caki-1 proliferation (Fig. 5A). PHDA1 knockdown in Caki-1 cells increased proliferation, but when both PDHA1 and SIRT5 were reintroduced, cell proliferation decreased significantly (Fig. 5B). Interestingly, overexpression of PDHA1^K351Q^ increased the proliferation of Caki-1 cells, but SIRT5 had no effect on this cell line. SIRT5 knockdown also significantly promoted proliferation (Fig. 5C and D) and migration (Fig. 5E and G), whereas SIRT5 inhibited cell migration through PDHA1 but did not reverse the PDHA1^K351Q^-mediated increase in cell migration (Fig. 5F). At the same time, ccRCC organoid models were established to confirm the potential clinical value of SIRT5. SIRT5 knockdown significantly increased cell proliferation in the PDOs generated from two different patients with ccRCC (Fig. 5H). These results supported the hypothesis that desuccinylation of PDHA1 by SIRT5 regulated metabolic pathways and inhibited cell proliferation, migration and invasion of ccRCC.

### SIRT5 correlates with PDHA1 hyposuccinylation and progression of ccRCC

**Fig. 6:**
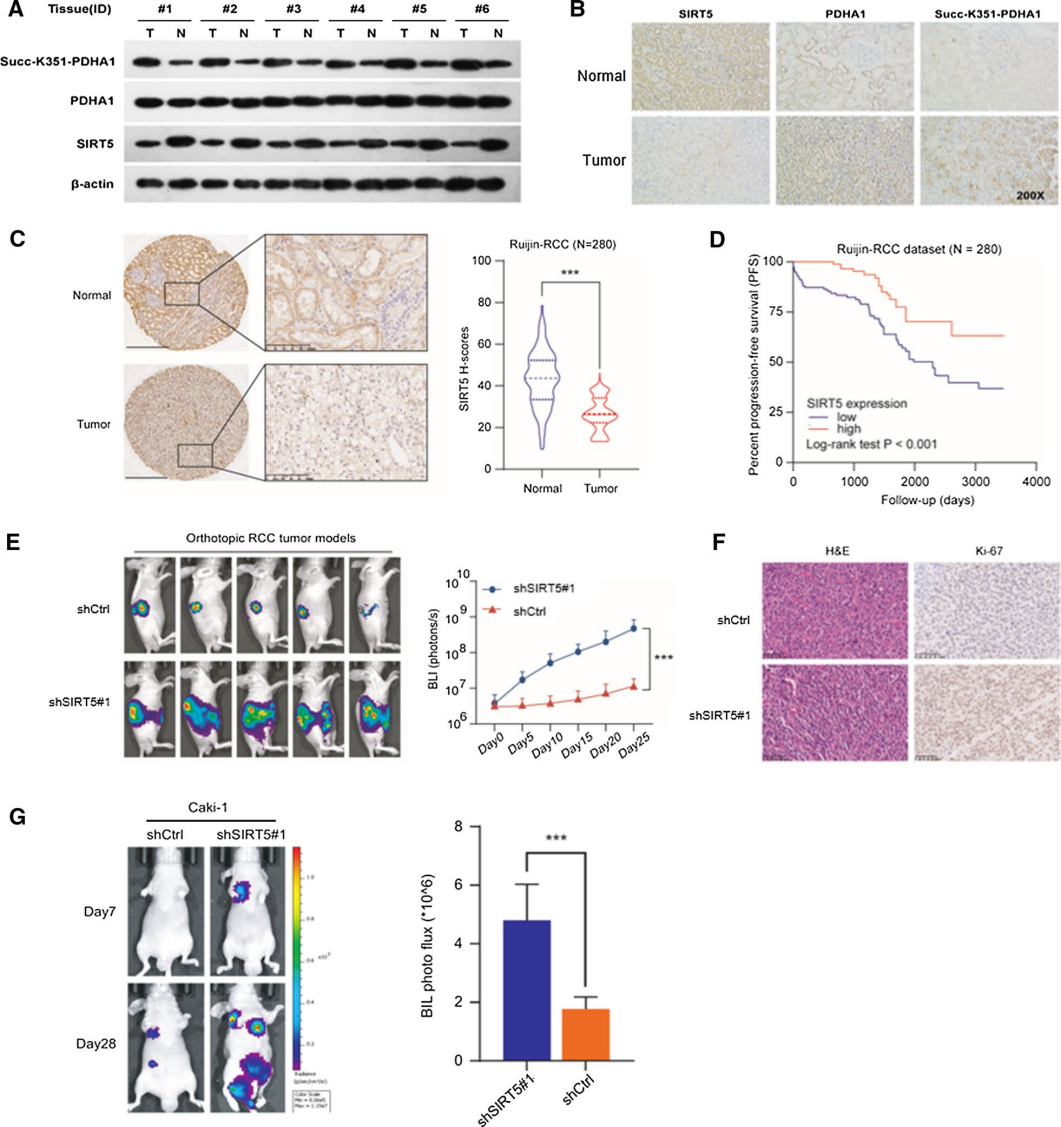
SIRT5 correlates with hyposuccinylation and progression of ccRCC. The levels of succinylated PDHA1 and SIRT5 were compared in 6 paired tumor and normal tissues using western blotting (**A**) and immunohistochemistry (**B**). **C** Representative images of immunohistochemical staining for SIRT5 protein in the Ruijin-ccRCC dataset. SIRT5 expression in tumor and normal tissues from the Ruijin-ccRCC dataset. **D** Kaplan–Meier analysis of PFS of patients stratified by SIRT5 expression in the Ruijin-ccRCC dataset. In vivo BLI (**E**) and HE staining (**F**) of an orthotopic model generated with WT and SIRT5-KD Luc-RENCA cells. **G** In vivo BLI of a lung metastasis model generated with WT and SIRT5-KD Luc-Caki-1 cells

Immunohistochemistry and Western blot assays were conducted using 6 paired ccRCC specimens to illustrate the differential expression of each marker in normal and tumor tissues. SIRT5 was expressed at low levels in tumor tissues, and levels of PHDA1 succinylated at K351 were observed in ccRCC samples (Fig. 6A and B). The results provided evidence that hypersuccinylation of PDHA1 promoted ccRCC progression, which was reversed by overexpression of SIRT5. Immunohistochemical staining and statistical analysis of the Ruijin-ccRCC cohort (n = 280) showed that SIRT5 expression was significantly decreased in ccRCC samples compared with normal tissues (Fig. 6C). The progression-free survival rate of patients with low SIRT5 expression was significantly higher than that of patients with high SIRT5 expression (*P* < 0.05) (Fig. 6D). The animal study showed that the tumor size of the SIRT5-KD group was significantly larger than that of the control group (Fig. 6E). HE staining and immunohistochemistry also showed that the percentages of Ki-67-positive cells were apparently increased in the SIRT5-KD group (Fig. 6F). SIRT5 knockdown significantly promoted metastatic processes compared with the control group in a lung metastasis model (Fig. 6G). Based on these results, SIRT5 suppressed ccRCC tumorigenesis by regulating the hyposuccinylation of PHDA1.

## Discussion

Most cancer cells exhibit an increased dependence on glycolysis to meet their energy demands, regardless of whether ample oxygen is available [6, 25]. PDC converts pyruvate into acetyl-CoA and links glycolysis and the TCA cycle [7–12]. Eukaryotic PDC is composed of E1, E2 and E3. Eukaryotic cells evolved hierarchical regulatory strategies through posttranslational modification, such as phosphorylation [16], acetylation [16, 21], succinylation [17, 18, 22, 24], glutarylation [26], malonylation [19, 27], and aminoacylation [28]. PDC activity was reported to be regulated by the phosphorylation of PDHA1, which is phosphorylated by PDK1-4 and dephosphorylated by PDP1-2 [6–13]. Deacetylation of K321 in PDHA1 by SIRT3 increases its activity [19, 20]. SIRT4, a mitochondria-targeted sirtuin, hydrolyses DLAT and diminishes PDH activity [23]. PDC has been recently reported to translocate into the nucleus and generate acetyl-CoA for histone acetylation [14], suggesting that the functions of PDC still require further investigation.

**Fig. 7:**
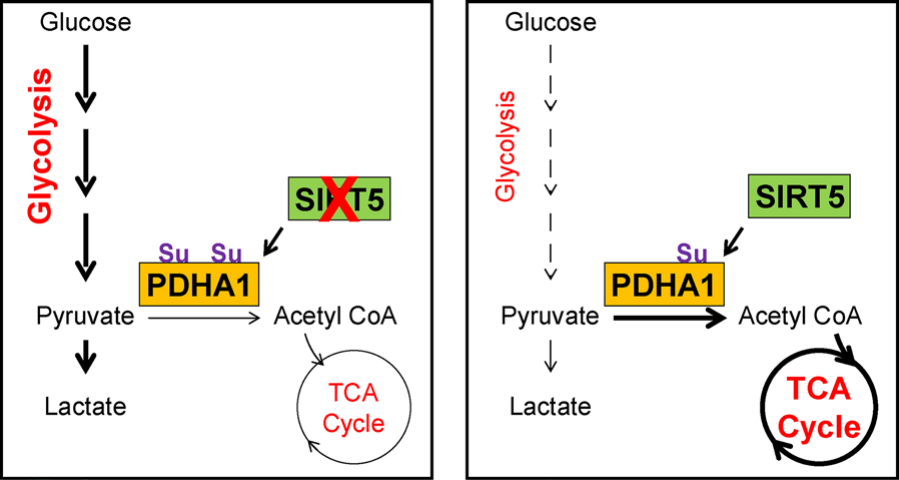
Schematic diagram of the indicated mechanisms by which SIRT5 reversed the Warburg effect

In the current study, we found that SIRT5 was significantly downregulated in ccRCC tissues compared with normal tissues and associated with a poor prognosis. SIRT5 deficiency significantly facilitated cell proliferation, migration, and invasion in vitro and promoted ccRCC tumorigenesis and metastasis in vivo*.* SIRT5 overexpression significantly decreased the oxygen consumption rate and inhibited cell proliferation and migration in ccRCC. The underlying mechanism showed that downregulated expression of SIRT5 in ccRCC resulted in hypersuccinylated PDHA1 at K351 and decreased PDC activity, thus accelerating the Warburg effect (Fig. 7). The results of our study showed that SIRT5 functioned as a tumor suppressor in ccRCC and correlated with metabolic reprogramming during tumor growth and metastasis by regulating the hyposuccinylation of PHDA1.

SIRT5, one of the three mitochondrial sirtuins, was reported to deacetylate and activate urate oxidase in the liver mitochondria of mice [29], but SIRT5 was reported to mediate posttranslational modifications of other proteins, such as desuccinylation [12, 13, 17, 19], glutarylation [26] and malonylation [19, 27]. SIRT5 desuccinylates and activates SOD1 to eliminate ROS [30], desuccinylates and activates PKM2 to block macrophage IL-1β production and prevent DSS-induced colitis [31], and desuccinylates and activates SHMT2 to drive cancer cell proliferation [32]. Recently, two papers published in Molecular Cell [20, 24] showed that SIRT5 desuccinylates PDHA1 and SHDB to positively or negatively regulate protein enzymatic activity. SIRT5 promotes melanoma cell survival partially through PDC [33].

SIRT5 has been previously reported as a master metabolic regulator, and its expression levels are significantly associated with the metabolic patterns and functions of various cell types [34]. SIRT5 not only maintains mitochondrial function through posttranslational modifications of mitochondrial proteins and enzymes but also modulates different pathways, including glucose oxidation, ketone body formation, fatty acid degradation, ammonia disposal, and redox homeostasis. Furthermore, SIRT5 appears to be a key determinant of metabolic rewiring in response to environmental stress [35].

Despite these impactful findings, our study has some limitations. First, we have not yet clearly determined how to define high and low expression of SIRT5, and further immunohistochemical studies in a large cohort are necessary to identify the optimal cut-off value. Second, since isocitrate dehydrogenase 2 and glucose 6-phosphate dehydrogenase, which are electron transport chain complexes, are regulated by SIRT5, a meaningful study would be to determine whether SIRT5 regulates metabolic reprogramming by targeting other related enzymes. Moreover, a systematic description of whether SIRT5 is associated with other clinical phenotypes, such as stem cells, angiogenesis, and tyrosine kinase inhibitor resistance, is needed. Future research based on a more comprehensive study design is needed to explore the underlying mechanism.

## Conclusions

Downregulated expression of SIRT5 in ccRCC accelerated the Warburg effect through PDHA1 hypersuccinylation and finally caused tumorigenesis and progression, which were reversed by SIRT5 overexpression. Our study suggested that SIRT5 may function as a potential target for ccRCC therapy.
